# AFM41a: A Novel PAD2 Inhibitor for Sepsis Treatment—Efficacy and Mechanism

**DOI:** 10.7150/ijbs.97166

**Published:** 2024-09-16

**Authors:** Tao Dong, Leonard Barasa, Xin Yu, Wenlu Ouyang, Liujiazi Shao, Chao Quan, Su He Wang, Jifeng Zhang, Morgan Salmon, Allan Tsung, Hasan B. Alam, Jianjie Ma, Paul R. Thompson, Yongqing Li

**Affiliations:** 1 Department of Surgery, University of Michigan Health System, Ann Arbor, MI, USA.; 2 Department of Physiology, Xuzhou Medical University, Xuzhou, Jiangsu, China.; 3 Program in Chemical Biology, Department of Biochemistry and Molecular Biotechnology, University of Massachusetts Chan Medical School, Worcester, MA 01605, USA.; 4 Department of Emergency Medicine, Second Affiliated Hospital, Zhejiang University School of Medicine, No. 88 Jiefang Road, Hangzhou, 310009, Zhejiang Province, China.; 5 Department of Metabolism and Endocrinology, The Second Xiangya Hospital, Changsha, China.; 6 Department of Anesthesiology, Beijing Friendship Hospital, Capital Medical University, Beijing, China.; 7 Department of Urology, The Xiangya Hospital, Changsha, China.; 8 Internal Medicine, Division of Allergy, University of Michigan Health System, Ann Arbor, MI, USA.; 9 Department of Internal Medicine, University of Michigan, Ann Arbor, MI, USA.; 10 Department of Cardiac Surgery, University of Michigan, Ann Arbor, MI, USA.; 11 Department of Surgery, Division of Surgical Science, University of Virginia, Charlottesville, VA 22903, USA.; 12 Department of Surgery, Northwestern University, Arkes Pavilion, 676 N St Clair St Ste 2320, Chicago, IL 60611, USA.

**Keywords:** AFM41a, macrophage polarization, autophagy, *Pseudomonas aeruginosa*

## Abstract

*Pseudomonas aeruginosa* (PA) infection can cause pneumonia and sepsis by activating peptidyl-arginine deiminase (PAD) and triggering the formation of neutrophil extracellular traps (NETs). Our previous research has elucidated the crucial role of PAD2 in regulating CitH3 production and NETosis signaling following bacterial infection. Therefore, targeting PAD2 with selective inhibitors holds promise for treating PA-induced sepsis. Here, we compare the structure and function of two PAD2 inhibitors, AFM32a and AFM41a, and investigate their biological effects in mice subjected with PA. We analyze their impact on PAD2 inhibition, macrophage polarization, and other host defense mechanisms against PA-induced sepsis utilizing both *in vivo* and *in vitro* approaches. Our findings demonstrate that both PAD2 inhibitors (AFM32a and AFM41a) and *Pad2* deficiency substantially enhance protection against PA-induced sepsis, with AFM41a showing superior efficacy over AFM32a. This protective effect is marked by improved survival rates, reduced bacterial growth in mice subjected to PA infection, and the promotion of M2 macrophage polarization coupled with enhanced autophagic activity. Our results advocate for targeting PAD2 as an effective strategy to bolster host defenses against PA infection. Utilizing AFM41a to promote M2 macrophage polarization and autophagy offers promising avenues for the treatment of PA infection and the improvement of sepsis outcomes.

## Introduction

Sepsis remains a major global health challenge, characterized by a dysregulated host response to infection, often leading to organ dysfunction and high mortality rates 1. *Pseudomonas aeruginosa* (PA) stands out as a common pathogen responsible for severe infections, including pneumonia, which can escalate into sepsis if untreated 2. The prevalence of PA in hospital-acquired pneumonia poses a heightened risk, particularly in immunocompromised individuals 3. Furthermore, PA's resistance to a broad spectrum of antibiotics presents a formidable obstacle in the search for effective therapies 4.

Macrophages play a vital role in orchestrating the immune response during PA infection, exhibiting a diverse range of phenotypical and functional characteristics 5. They possess the remarkable ability to adapt their functional profiles based on environmental cues 6. Macrophages can broadly be classified into two main subsets: 1) classically activated M1 macrophages, which are involved in pro-inflammatory responses and pathogen elimination, and 2) alternatively activated M2 macrophages, known for their anti-inflammatory functions and tissue repair capabilities 7. The balance between M1 and M2 macrophage polarization is critical for an efficient immune response and ultimately influences the outcome of infectious diseases, including sepsis 8.

Protein citrullination, the conversion of arginine into citrulline 9 is a biochemical process mediated by a family of calcium-dependent enzymes known as peptidylarginine deiminases (PADs). PADs, encompassing five isozymes (PAD1-4 and PAD6)^10^, play integral roles in various physiological processes, including immune responses, cell signaling, and gene expression regulation. PAD2, a member of the PAD isozyme family, exhibits prominent expression in monocytes, macrophages, and neutrophils 11, PAD2 is aberrantly increased in numerous diseases including rheumatoid arthritis 12, multiple sclerosis 13, and neurodegenerative disorders 14. Previous investigations have highlighted the crucial role of PAD2-catalyzed citrullination in diverse modes of immune cell death, such as ETosis 15, 16 and pyroptosis 17, which are believed to have a profound impact on the pathogenesis of sepsis and other inflammatory conditions.

Our recent study demonstrated that the absence of the *Pad2* gene improves survival in a cecal ligation and puncture (CLP) mouse model of sepsis. Furthermore, these investigations have revealed that PAD2 knockout in macrophages reduces Caspase-1 mediated pyroptosis in PA-induced sepsis, whereas PAD4 depletion in macrophages increases Caspase-1 mediated pyroptosis in the same mouse model 18,19. However, the specific role of the novel PAD2 inhibitor AFM41a in sepsis, as well as the involvement of PAD2 in macrophage polarization during PA infection-induced sepsis, remains poorly understood.

In the present study, we aim to explore the connection between PAD2 activity and macrophage polarization, employing both *in vivo* and *in vitro* approaches. We will examine the role of autophagy, a cellular process crucial for regulating immune responses 20 and maintaining cell homeostasis 21, in observed macrophage polarization. Understanding the interplay between PAD2 and macrophage polarization could offer valuable insights into the immunopathogenesis of sepsis and potentially identify therapeutic targets for enhancing host defense mechanisms and improving outcomes in PA-induced sepsis. Specifically, our study seeks to assess the impact of the novel PAD2 inhibitor AFM41a and *Pad2* deficiency on macrophage polarization during PA infection-induced sepsis.

## Materials and Methods

### Animals

WT male C57BL/6J and* Pad2^-/-^* mice (8-10 weeks old) were used in this study. WT mice were purchased from the Jackson Laboratory (Bar Harbor, ME, USA). *Pad2*^-/-^ mice were kindly provided by Dr Scott Coonrod (Cornell University, Ithaca, New York, USA). The mice were housed in a specific pathogen-free environment with a controlled temperature of 23±1.5°C and relative humidity of 70±20%. Experimental protocols and animal care methods were reviewed and approved by the University of Michigan Institutional Animal Care and Use Committee and performed according to their guidelines.

### Cells

Mice were sacrificed, and the thoracic cavity and trachea were carefully dissected. A small incision was made in the trachea using a 1-mL syringe with an angiocath (BD Biosciences, Franklin Lakes, NJ), and the bronchoalveolar lavage (BAL) fluid was collected into a sterile tube. The lungs were lavaged three times with 1 mL of phosphate-buffered saline (PBS) containing 1% fetal bovine serum (FBS, Life Technologies, Grand Island, NY). The recovered BAL fluid was centrifuged at 500 g for 5 minutes at 4 °C to obtain the cell pellets. The cell pellets were resuspended in RPMI 1640 medium (Life Technologies) supplemented with 10% heat-inactivated fetal bovine serum (Invitrogen) and incubated on a culture plate for 1.5 hour at 37 °C/5% CO_2_ incubator to allow alveolar macrophages attachment. Non-adherent cells were then removed by washing with normal saline. The distribution of macrophage population was analyzed by using flow cytometry (**Figure S6**).

Human monocytic THP-1 cells were maintained in RPMI 1640 (Life Technologies) culture medium supplemented with 10% heat-inactivated fetal bovine serum (Invitrogen) at 37 °C/5% CO_2_ incubator. The culture medium was further supplemented with 10 mM Hepes (Gibco, #15630-056), 1 mM pyruvate (Gibco, #11360-039), 2.5 g/l D-glucose (Merck), and 50 pM ß-mercaptoethanol (Gibco; 31350-010). To differentiate THP-1 monocytes into macrophages, cells were incubated with 150 nM phorbol 12-myristate 13-acetate (PMA, Sigma, P8139) for 24 hours, followed by an additional 24 hours of incubation in RPMI medium. These primary cells and cell lines provided essential tools for our study, allowing us to investigate the role of macrophages and immune responses in the context of our research on PA-induced sepsis.

### Pulmonary infection model

The bacterial strains used in this study, PA WT strain 19660 from ATCC (Manassas, Virginia, USA) and PA Xen-41 from PerkinElmer-Caliper (Waltham, MA, USA), were prepared in PBS at concentrations of 8.25×10^7^ colony-forming units (CFU)/mL. To establish the PA pneumonia-induced sepsis model, mice aged 8-10 weeks were subjected to the following procedure following established protocols 22. PA 19660 or PA Xen-41 solution was prepared in PBS at concentrations of 8.25×10^7^ colony-forming units (CFU)/mL. The mice were anesthetized with ketamine (100 mg/kg body weight) and xylazine (20 mg/kg), and then held vertically. A total of 30 μL of PA solution (15 μL in each nostril) was administered, resulting in a final bacterial load of 2.5×10^6^ CFU. Sham control animals were inoculated with sterile PBS. For non-survival studies, mice were euthanized by CO_2_ at 24 hours after inoculation. Serum, BALF, and organs were collected and stored at -80°C for further analysis. In survival studies, groups of mice (n=8/group) were monitored for a period of 10 days, and those found to be in a moribund state were euthanized with CO_2_. The endpoint of observation was also considered as a time for euthanization. This model allowed us to investigate the progression of PA pneumonia-induced sepsis and its impact on the mice over time, providing valuable data for our study on the effects of *Pad2* deficiency and inhibition on host defense and bacterial dissemination during PA-induced sepsis.

### *In vivo* imaging

Throughout the course of post-infection, PA Xen-41 infected mice were subjected to whole-body imaging using an IVIS XRII system (PerkinElmer, Waltham, MA). The imaging process followed the user guides provided by PerkinElmer-Caliper, ensuring accurate and standardized data acquisition. This imaging technique allowed us to monitor the dynamic distribution and progression of the PA Xen-41 infection in the mice over time, providing valuable insights into the bacterial dissemination process during the study 9.

### Infection experiments

Bacterial cultures were prepared by overnight growth in Luria-Bertani (LB) broth at 37°C with shaking. The bacterial cells were then collected by centrifugation at 5,000 g and resuspended in 10 mL of fresh LB broth. To ensure consistent vitality, the bacterial density was adjusted to an optical density (OD) of 0.5-0.6 at 600 nm (OD600) in a spectrophotometer. (Note: 1 OD at 600 nm corresponds to approximately 1×10^9^ cells/mL). Mammalian cells were cultured overnight in RPMI 1640 medium supplemented with 10% fetal bovine serum and antibiotics. Prior to infection, the cells were washed once with PBS and switched to serum-free and antibiotic-free media. For the infection experiments, mammalian cells were infected with a bacterial load 20 times higher than the number of cells (bacteria-to-cell ratio of 100:1) for the specified time points. This ensured an effective and consistent infection process for the indicated duration. These infection experiments allowed us to investigate the interactions between the bacterial pathogen and mammalian cells, providing valuable data for our study on host-pathogen interactions during our research on PA-induced sepsis.

### 3-(4,5-Dimethylthiazol-2-yl)-2,5-dimethyltetrazolium bromide (MTT) assay

Cells were treated as described previously, and an equal amount of MTT dye was added to the cell culture. Following a 1-hour incubation period, the reaction was halted using a stop solution, and the mixture was left at room temperature overnight to ensure complete dissolution of formazan crystals. Subsequently, the absorbance at 560 nm was measured using a multi-scan plate reader, allowing for the quantification of superoxide anion concentration.

### Phagocytosis assay

The pHrodo Red E. coli BioParticles (Thermo Fisher Scientific, P35361) were suspended in living imaging solution at a concentration of 1 mg/mL and sonicated to ensure a homogeneously dispersed suspension. Next, the cells were incubated with the pHrodo Red E. coli BioParticles for 1-1.5 hours at 37°C. To visualize the phagocytosis process, representative images were captured using a fluorescence microscope (Olympus, DP70). For each group, six microscopic fields were randomly selected for observation.

### Quantitative Real-Time PCR

Quantitative Real-Time PCR (qRT-PCR) was performed using the CFX Connect Real-Time PCR Detection System (Bio-Rad, Hercules, CA) and gene-specific primers (see Supplementary Information Table S1 for primer details, synthesized in Integrated DNA Technologies, Coralville, IA). To analyze relative transcript levels, initial normalization was done using the cycle threshold (CT) values to the housekeeping gene GAPDH. Subsequently, the data was further normalized to the indicated control using the 2^-ΔΔCT^ method.

### Western blot

Electrophoresis on 10% sodium dodecyl sulfate-polyacrylamide gels were used to separate the cellular proteins, which were then transferred to polyvinylidene difluoride membranes for immunoblotting. The membranes were blocked for 1 hour at room temperature with 5 % skimmed milk in PBS, supplemented with 0.1% Tween 20. The blots were incubated overnight with diluted primary antibodies (iNOS, PA1-036, Invitrogen; Arg-1, PA5-85267, Invitrogen; CD206, MA5-32498, Invitrogen; LC3A/B, 4108s, Cell Signaling Technology; Beclin-1, 3738s, Cell Signaling Technology; P62, D1Q5S, Cell Signaling Technology; PAD2, 12110-AP, Proteintech; CitH3, ab5103, Abcam and β-actin, 4970S, Cell Signaling Technology), and then incubated for 1 h with a secondary antibody (Invitrogen, USA) at room temperature. Subsequently, the membranes were again washed three times for 10 min each, and the antigen-antibody complexes were visualized using enhanced chemiluminescence (Bio-Rad, Hercules, CA). The specific protein bands were imaged using a Luminescent image analyzer (Thermo Fisher Scientific, USA).

### ELISA

TNF-α, IL-6, and IL-10 levels in cell culture supernatants and BALF were measured using an ELISA kit (Thermo Fisher Scientific, USA) according to the manufacturer's instructions**.**

### Immunofluorescence

Cells were subjected to blocking and then incubated with primary antibodies against iNOS, PA1-036, Invitrogen; Arg-1, PA5-85267, Invitrogen; CD206, MA5-32498, Invitrogen; and LC3B, 43566s, Cell Signaling Technology overnight at 4°C. Subsequently, the sections were rinsed twice to remove any unbound antibodies and then incubated with the corresponding secondary antibody for 1 hour at room temperature. The secondary antibody used in this study was obtained from Invitrogen (DAKO, USA).

### Flow cytometry

Cells were washed three times with PBS and then centrifuged at 500g for 5 minutes. The cells were then resuspended in 500 μl of PBS. For specific cell surface marker analysis, CD45 antibody (103128, BioLegend) with Alexa Fluor 700 and F4/80 antibody (11-4801-82, Invitrogen) with FITC and CD11c (566504, BD) with PB were individually added to the cells. Following this, the cells were incubated in the dark for 30 minutes at room temperature. After the incubation period, the cells were washed twice with PBS and filtered using a 40 μm filter to prepare for flow cytometry analysis. To investigate intracellular markers of macrophage activation, iNOS (17-5920-82, Invitrogen) and Arg-1 (IC5868P, R&D) were stained using the Staining Intracellular Antigens for Flow Cytometry kit (00-5521-00, Thermo Fisher Scientific), following the user guidelines provided by the company.

### Hematoxylin and eosin staining

Lung injury was evaluated through pathological analysis. Lung tissues were fixed in formalin, embedded in paraffin wax, and sectioned at a thickness of 4 µm. The sections were then stained with hematoxylin and eosin (H&E) to visualize the tissue morphology. A blinded pathologist assessed the stained sections, and lung injury scores were determined using Suzuki's method. This scoring system allowed for an objective evaluation of the degree of lung injury, ensuring consistent and unbiased results.

### Transmission Electron Microscopy (TEM) Sample Preparation and Analysis

The preparation of samples for TEM followed the method outlined by Di Cristina *et al.*, 2017 23. To prepare the samples for TEM, cells were washed three times with cold PBS, then fixed with 2.5% glutaraldehyde in 0.1 M sodium cacodylate buffer (pH 7.4) for 1 hour at room temperature. The fixed monolayers were carefully scraped to detach large cell sheets, which were then transferred to microcentrifuge tubes. The samples were centrifuged at 1500 g for 10 minutes at room temperature, followed by three washes with 0.1 M sodium cacodylate buffer (pH 7.4). The samples were stored in the same buffer at 4°C until further processing. For TEM examination, the samples were prepared according to the protocol by Coppens and Joiner, 2003, and observed using a JEOL JEM-1400+ transmission electron microscope operated at 60 kV.

### Statistical analysis

The statistical analysis was conducted using GraphPad Prism 7 software. Mantel-Cox tests were utilized to analyze the survival curve and assess differences in survival rates among the experimental groups. For comparisons involving three or more groups, we employed one-way analysis of variance (ANOVA) with Bonferroni's multiple comparison test to determine significant differences between individual groups. The Student's t-test, a parametric test, was applied for comparisons between two groups after confirming that the data followed a normal distribution. All data are expressed as mean ± standard error of the mean (SEM) to represent the central tendency and the precision of the measurements. A threshold of P < 0.05 was considered statistically significant, indicating the presence of meaningful differences between the compared groups.

## Results

### PAD2 inhibition decreases susceptibility and improves survival against *Pseudomonas aeruginosa* infection

**Figure 1:**
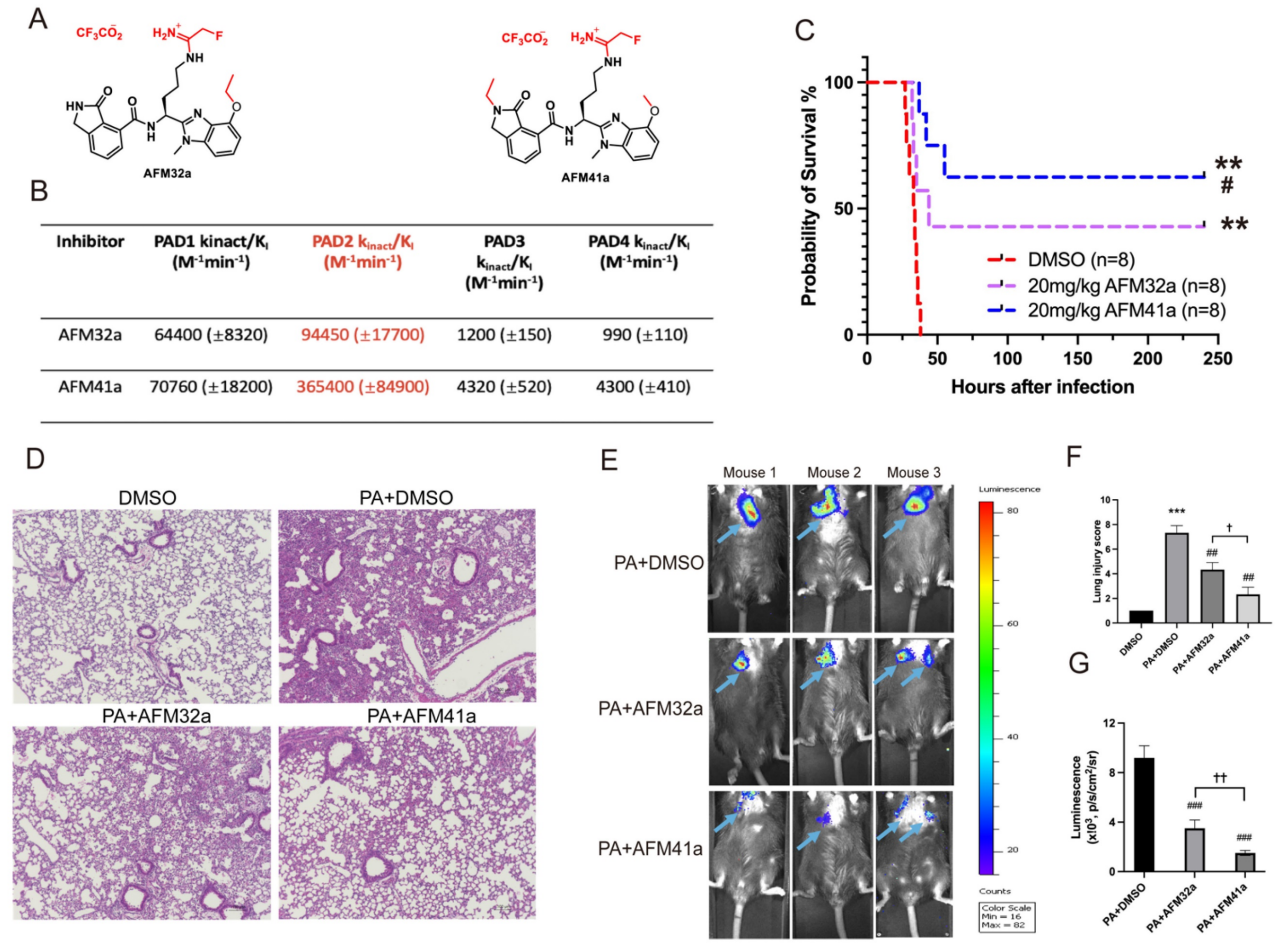
** PAD2 inhibitors decreased susceptibility and mortality against *Pseudomonas aeruginosa* (PA) infection.** (A-B) Structures and K_inct_/K_I_ values for PAD2 inhibitors AFM32a and AFM41a. (C) WT mice were intranasally challenged with 2.5×10^6^ CFU PA 19660/mouse and subsequently administered a single dose of AFM32a (20 mg/kg of body weight) or AFM41a (20 mg/kg of body weight) or an equivalent dose of DMSO 0.5 hours after infection. Survival rates were monitored for 10 days (n=8/group), and analysis was conducted using Mantel-Cox test. (D) Lung tissue samples were collected 24 hours after PA infection and subjected to H&E staining. (E) Whole animal imaging of bioluminescence was obtained using IVIS XRII system 24 hours after mice challenged with 2.5×10^6^ CFU PA Xen-41/mouse (arrows indicating PA spread regions). (F) Lung injury scores. (G) Quantitative analysis of bioluminescence. *p<0.05 vs DMSO; **p<0.01 vs DMSO; ***p<0.001 vs DMSO; #p<0.05 vs 20mg/kg AFM32a; ##p<0.01 vs PA+AFM32a; ✚ p<0.05 vs PA+AFM32a.

In our previous study, we established that *Pad2* deficiency provides protection against sepsis induced by PA pneumonia 19. Consequently, targeting PAD2 with selective inhibitors emerges as a promising avenue for managing PA-induced sepsis. Employing a medicinal chemistry approach, we previously described the development of several PAD2 selective inhibitors including AFM32a, and AFM41a 24 .The compounds covalently modify the active site cysteine (C647) in PAD2 to irreversibly inhibit the activity of this enzyme. Based on a comparison of their *k*_inact_/*K*_I_ values, the best potency measure for an irreversible inhibitor, AFM41a is 4-fold more potent than AFM32a and 85-fold selective for PAD2 over PAD4 24 (**Figure 1A & Figure 1B**).

To investigate the role of PAD2 inhibitors (AFM32a and AFM41a) in PA-induced sepsis, we established an acute pneumonia model by intranasally instilling PA (2.5×10^6^ CFU/mouse) into Wild Type (WT) mice. Subsequently, WT mice were treated with either dimethyl sulfoxide (DMSO; control) or AFM32a and AFM41a, administered 30 min after PA challenge. As illustrated in **Figure 1C**, both AFM32a and AFM41a improved survival rates, with AFM41a demonstrating higher efficacy than AFM32a. H&E staining revealed that compared to the DMSO treatment group, PAD2 inhibitor treatment efficiently decreased lung injury, with AFM41a exhibiting greater efficiency than AFM32a (**Figure 1D & Figure 1F**).

To directly visualize the *in vivo* infection process, we administered PA Xen-41, an engineered bacterium emitting bioluminescence for imaging, to mice at the same dosage. Remarkably, at 24 hours post-infection, both AFM32a and AFM41a treatments resulted in a narrower area of bioluminescence in the thoracic cavity, with AFM41a-treated mice exhibiting the narrowest area of bioluminescence (**Figure 1E & Figure 1G**).

Furthermore, to validate the role of PAD2 in susceptibility to PA infection, we observed and compared the patterns of bacterial dissemination between *Pad2^-/-^* and WT mice. At 24 hours post-infection, *Pad2^-/-^* mice displayed a narrower area of bioluminescence in the thoracic cavity, which subsequently expanded over time. Conversely, WT mice exhibited a broader distribution of bioluminescence (**Figure S1**).

### PAD2 inhibition promotes macrophage M2 polarization upon PA infection

In our previous study, we provided evidence that *Pad2* deficiency in mice challenged with PA led to an augmented presence of macrophages in the bronchoalveolar lavage fluid (BALF) compared to PA-challenged WT mice. This increase was attributed to a reduction in pyroptosis, suggesting a crucial role for alveolar macrophages in *Pad2* deficiency-mediated protection against PA-induced lung injury 19. Our current investigation aimed to elucidate the potential association between *Pad2* deficiency's protective effects and macrophage polarization.

To explore the impact of PAD2 inhibitors on macrophage polarization in human macrophages, we employed THP-1 macrophage as our experimental model. THP-1 macrophages were exposed to PA at a multiplicity of 100 at different time points: 0, 0.5 hour, 1 hour, 1.5 hour, 2 hour, and 3 hour. Our data revealed that the expression of PAD2 and CitH3 increased from 0.5 hour after PA infection, with cells undergoing destruction and death from 1.5 hours post-infection **(Figure S2 & Figure S3)**. As a result, for subsequent experiments, we selected a 1-hour infection time for THP-1 macrophages with PA.

**Figure 2:**
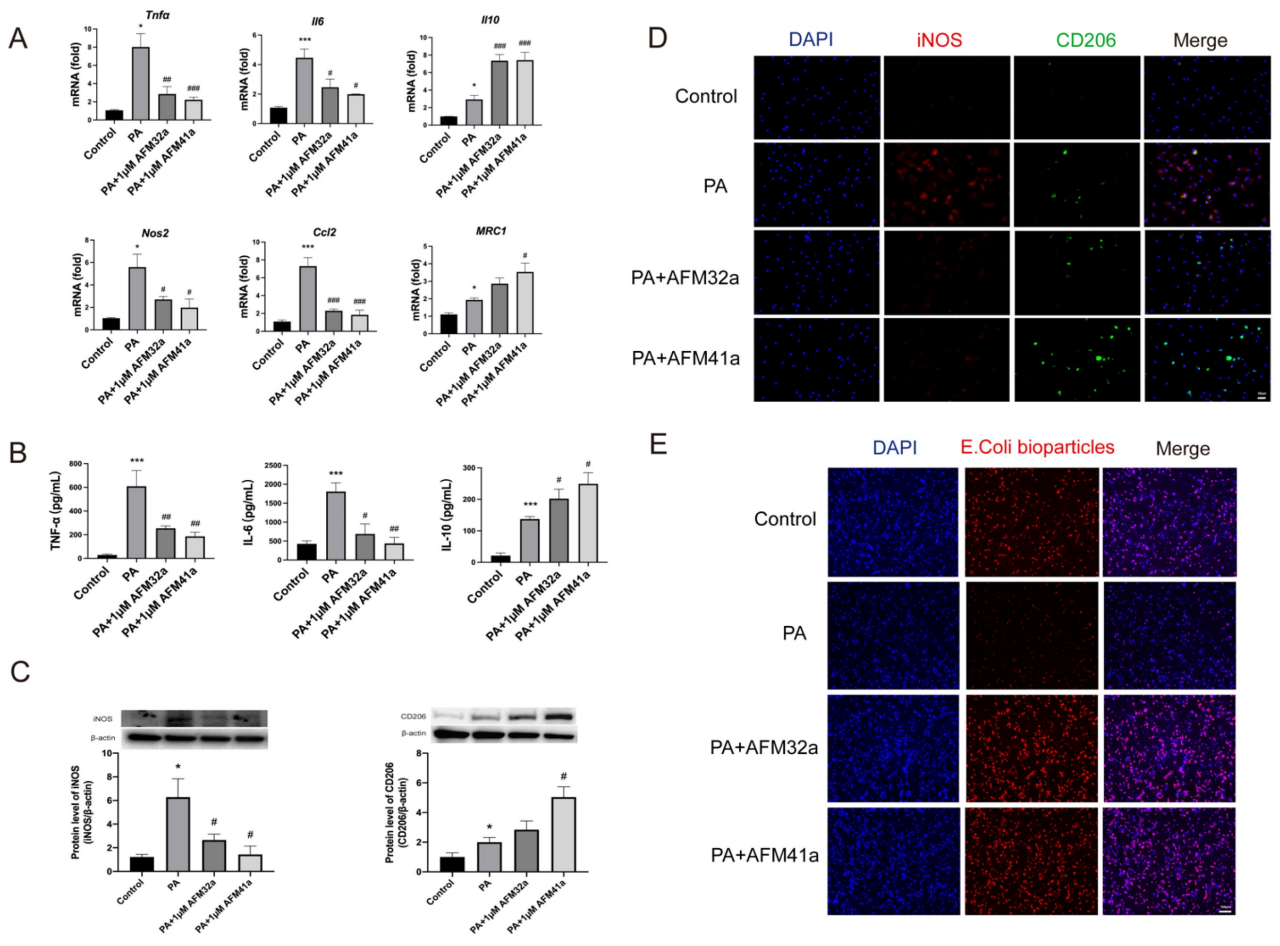
** PAD2 inhibitors promoted THP-1 macrophage towards M2 polarization and increased phagocytosis.** THP-1 cells were treated with PMA to induce differentiation for 22-24 hours prior to infection with* Pseudomonas aeruginosa* (PA) at a multiplicity of 100. Cells were then pre-treated with or without 1 µM AFM32a or AFM41a for 24 hours, then treated with or without PA for 1 hour. (A) The expression of classical cytokine genes (*Tnfα, Il6, and Il10*) and macrophage polarization signature genes (*Nos2, Ccl2, and Mrc1*) was quantified by qRT-PCR. (B) The concentrations of TNF-α, IL-6 and IL-10 were determined in cell culture supernatant. (C) Protein expression levels of iNOS and Arg-1 were measured using Western blot analysis. (D) Cells were then fixed and stained for iNOS (red), CD206 (green), and DAPI (blue). (E) Cells were then pre-treated with or without 1 µM AFM32a or AFM41a for 24 hours, then treated with or without PA for 1 hour. The phagocytic capacity of cells was measured using pHrodo Red E. coli BioParticles. Data are representative of three independent experiments expressed as means ± SEM. *p < 0.05 vs Control; ***p < 0.001 vs Control; #p < 0.05 vs PA; ##p < 0.01 vs PA; ###p < 0.001 vs PA.

We treated THP-1 macrophages with both AFM32a and AFM41a (**Figure 2A**) at various time points and concentrations. As depicted in **Figure S4**, the most efficient PAD2 inhibitors were observed at 1 µM concentration, administered 24 hours prior to infection. Additionally, in comparison to the Cl-amidine treatment group, both AFM32a and AFM41a treatment groups showed no effect on cell viability **(Figure S5)**. Consequently, we determined that a concentration of 1 µM for either AFM32a or AFM41a, administered 24 hours before infection, is the optimal choice for subsequent experiments.

Next, the expression levels of classic cytokine genes and macrophage polarization signature genes were measured by using qRT-PCR. The results indicated a significant decrease in the expression of pro-inflammatory cytokine genes (*Tnf-α, Il-6*) and M1 macrophage signature genes (*Nos2, Ccl2*) upon treatment with both AFM32a and AFM41a. Conversely, the expression of the anti-inflammatory gene (*Il-10*) and M2 macrophage signature gene (*Mrc1*) showed a significant increase in both AFM32a and AFM41a treatment groups** (Figure 2A)**. These findings were further supported by TNF-α, IL-6, and IL-10 ELISA analysis of cell culture supernatant** (Figure 2B)**.

To further validate the impact of PAD2 inhibition on THP-1 macrophage polarization, we employed western blot and immunofluorescence to evaluate macrophage phenotypes, particularly M1 markers (iNOS) and M2 markers (CD206). As depicted in **Figure 2C & Figure 2D**, the level of iNOS was reduced, while the level of CD206 was increased upon treatment with both AFM32a and AFM41a. These results indicate that both AFM32a and AFM41a effectively reduced M1 polarization and promoted M2 polarization. Notably, AFM41a exhibited greater efficacy in this regard compared to AFM32a. Additionally, we assessed the effect of AFM32a and AFM41a treatment on the phagocytic capacity of THP-1 macrophages. The results demonstrated that both AFM32a and AFM41a effectively increased phagocytosis, with AFM41a exhibiting greater efficacy in enhancing the phagocytic activity of THP-1 macrophages **(Figure 2E)**. These observations collectively support the notion that PAD2 inhibition in THP-1 macrophages results in a shift towards an anti-inflammatory M2 phenotype, accompanied by enhanced phagocytic activity, potentially contributing to the improved host defense against PA infection.

### *Pad2* deficiency promotes alveolar macrophages towards M2 polarization during PA infection

**Figure 3:**
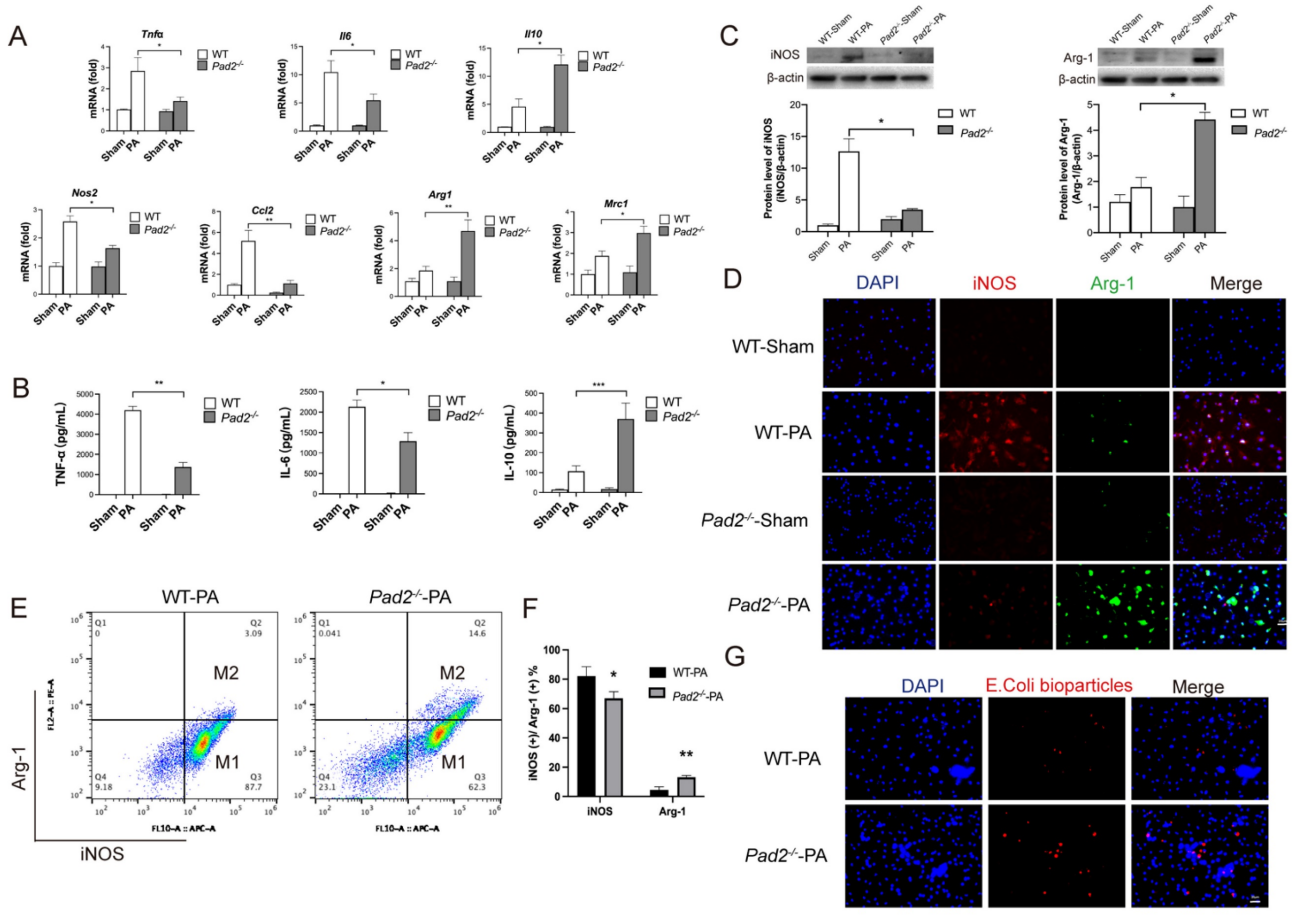
***Pad2* deficiency promoted Alveolar macrophages (AMs) towards M2 polarization and increased phagocytosis.** The *Pad2^-/-^* and WT mice were infected with 2.5 X 10^6^ CFU *Pseudomonas aeruginosa* (PA) 19660/mouse for 24 hours. (A) AMs from *Pad2^-/-^* and WT mice were lysed, and the expression of classical cytokine genes (*Tnfα, Il6, and Il10*) and macrophage polarization signature genes (*Nos2, Ccl2, Mrc1, and Arg1*) was quantified by qRT-PCR. (B) BALF was collected from both groups of mice, and the levels of inflammation cytokines (TNF-α, IL-6, and IL-10) were determined using ELISA. (C) AMs were isolated from *Pad2^-/-^* and WT mice, cell lysates from AMs were analyzed by western blotting to assess the protein levels of macrophage polarization markers, iNOS, and Arg-1. (D) AMs were subjected to immunofluorescence staining to evaluate the expression of macrophage polarization markers, iNOS, and Arg-1. (E) Isolated AMs from *Pad2^-/-^* and WT mice were analyzed by flow cytometry to quantify the distribution of M1 and M2 macrophage populations. (F) Quantification of the proportion of M1 and M2 macrophages. (G) The phagocytic capacity of AMs was measured using pHrodo Red E. coli BioParticles. Data are representative of three independent experiments expressed as means ± SEM. *p < 0.05; **p < 0.01; ***p < 0.01.

To further confirm the potential association between *Pad2* deficiency and macrophage polarization, alveolar macrophages were isolated from both WT and *Pad2^-/-^* mice after 24 hours of PA infection. We assessed the expression levels of classic cytokine genes and macrophage polarization signature genes using qRT-PCR. The results demonstrated significantly higher expression of pro-inflammatory cytokine genes (*Tnf-α*, *Il-6*) and M1 macrophage signature genes (*Nos2, Ccl2*) in WT alveolar macrophages compared to *Pad2^-/^*^-^ alveolar macrophages (**Figure 3A**). Conversely, the expression of the anti-inflammatory gene (*Il-10*) and M2 macrophage signature genes (*Mrc1 and Arg1*) was significantly higher in *Pad2^-/^*^-^ alveolar macrophages (**Figure 3A**). To further corroborate these findings, we performed enzyme linked immunosorbent assay (ELISA) analysis for TNF-α, IL-6, and IL-10 on BALF collected from the lungs of PA-infected WT and Pad2^-/-^ mice (**Figure 3B**). Our results strongly support a potential association between *Pad2* deficiency and macrophage polarization.

Further validating the influence of *Pad2* deficiency on macrophage polarization, we utilized western blot and immunofluorescence to assess macrophage phenotypes, specifically M1 markers (iNOS) and M2 markers (Arg-1) (**Figure 3C & Figure 3D**). The results confirmed reduced iNOS levels and elevated Arg-1 levels in alveolar macrophages of *Pad2^-/^*^-^ mice in comparison to WT mice.

Subsequently, we performed flow cytometry to analyze the polarization of alveolar macrophages following PA infection. *Pad2* deficiency led to a significant decrease in the population of iNOS-positive cells and a notable increase in the population of Arg-1-positive cells **(Figure 3E & 3F)**. Furthermore, we measured the phagocytic activity of alveolar macrophages from both WT and *Pad2^-/^*^-^ mice, demonstrating a significantly higher phagocytosis level in *Pad2^-/^*^-^ alveolar macrophages compared to WT alveolar macrophages **(Figure 3G)**. Taken together, our comprehensive analyses indicate that *Pad2* deficiency is associated with macrophage polarization, showing a shift towards the M2 phenotype, which may contribute to the macrophage phagocytosis and protective effects observed in PA-induced lung injury.

### Autophagy drives M2 macrophage polarization upon PA infection under PAD2 inhibition

Given the established involvement of autophagy in macrophage polarization regulation, and a reduction in autophagy levels compromise the elimination of PA within macrophages 25, 26. Based on these findings, we postulate that the augmented M2 macrophage polarization associated with *Pad2* deficiency in PA infection-induced sepsis is, at least in part, attributed to heightened autophagy in mutant alveolar macrophages. Initially, we assess the influence of autophagy on THP-1 macrophage polarization during PA infection while inhibiting PAD2. THP-1 macrophages were exposed to PA at various time points (0, 0.5h, 1h, and 2h), and our data revealed a significant increase in autophagy levels at 1 hour after PA infection (**Figure S7**).

**Figure 4:**
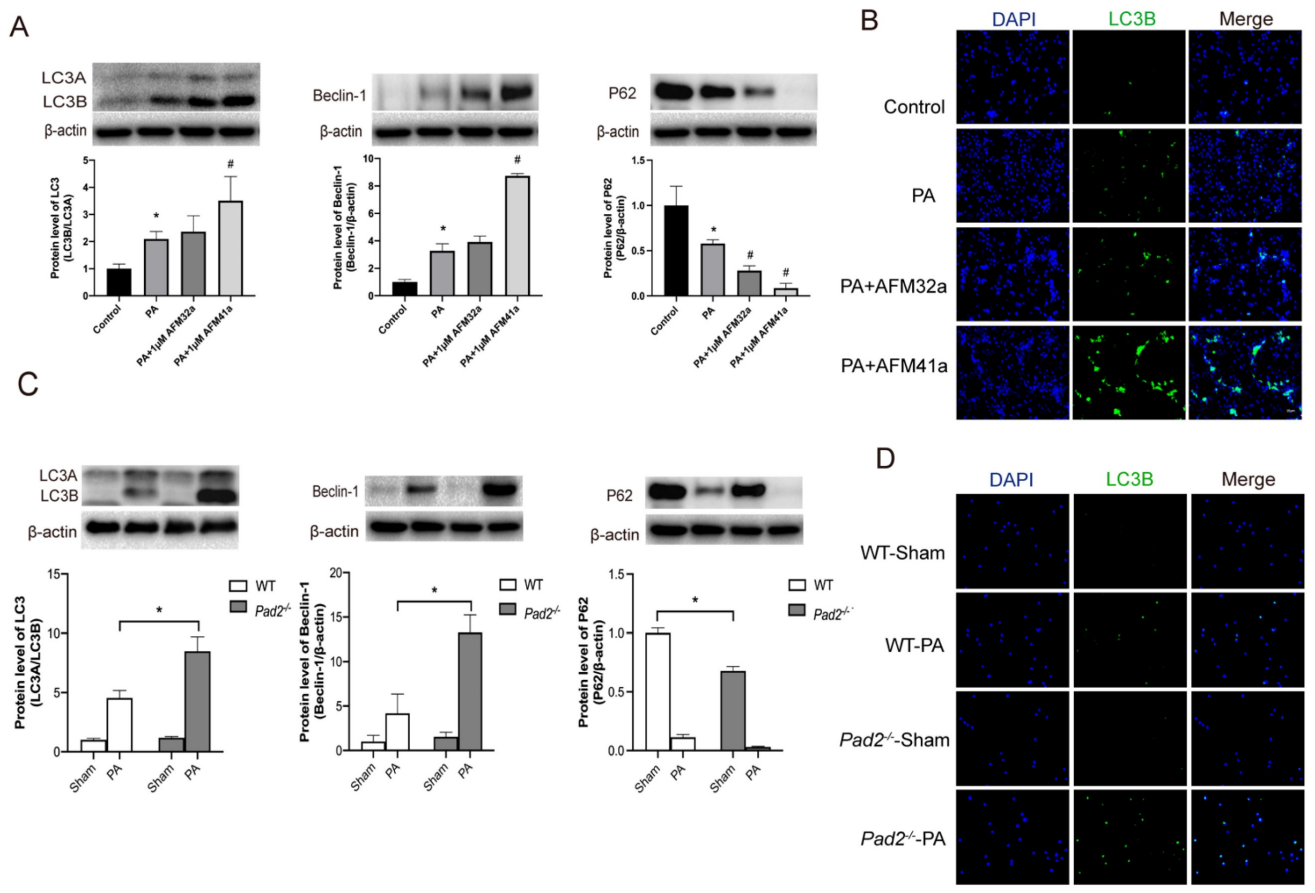
***Pad2* deficiency and PAD2 specific inhibitors increased autophagy level.** (A-B) THP-1 cells were treated with PMA to induce differentiation for 22-24 hours prior to infection with *Pseudomonas aeruginosa* (PA) at a multiplicity of 100. Cells were then pre-treated with or without 1 µM AFM32a or AFM41a for 24 hours, then treated with or without PA for 1 hour. Protein expression levels of LC3B, Beclin-1, and P62 were measured using immunofluorescence and western blot analysis. (C-D) The *Pad2^-/-^* and WT mice were infected with 2.5×10^6^ CFU PA 19660/mouse for 24 hours. Alveolar macrophages (AMs) of WT and *Pad2^-/-^* mice were analyzed by immunofluorescence and western blotting to assess the protein levels of autophagy related proteins, LC3, Beclin-1 and P62. Data are representative of three independent experiments expressed as means ± SEM. *p < 0.05 vs Control; #p < 0.05 vs PA.

**Figure 5:**
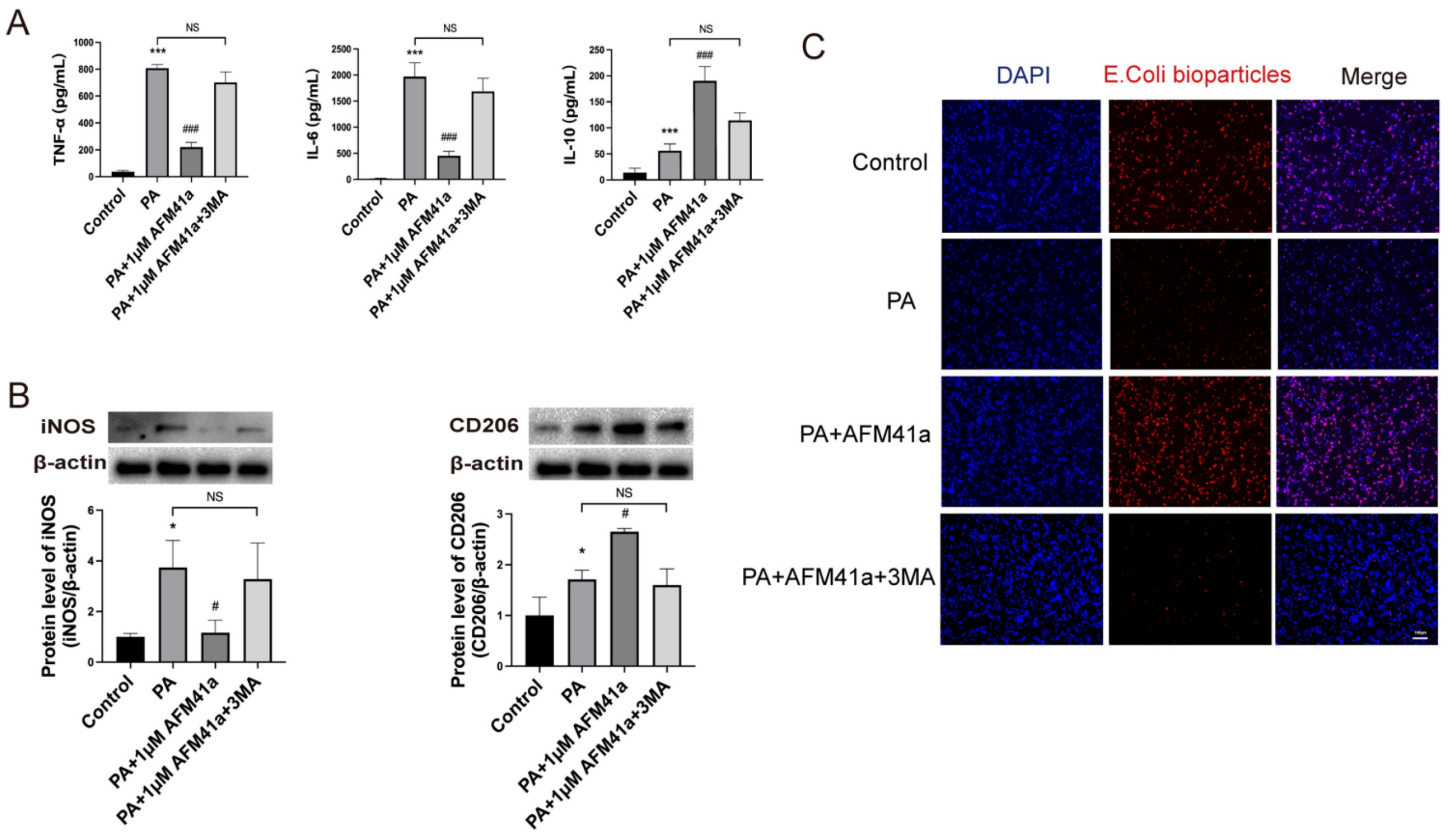
** PAD2 specific inhibitors promoted THP-1 macrophage towards M2 polarization through autophagy.** THP-1 cells were treated with PMA to induce differentiation for 22-24 hours prior to infection with *Pseudomonas aeruginosa* (PA) at a multiplicity of 100. Cells were then pre-treated with or without 1 µM AFM32a or AFM41a for 24 hours, then treated with or without PA for 1 hour. (A) Cells were treated with 1 µM AFM41a in combination with 5 µM 3MA, and the concentrations of TNF-α, IL-6, and IL-10 were determined in the cell culture supernatant. (B) Cells were treated with 1 µM AFM41a in combination with 5 µM 3MA, and the expression of iNOS and CD206 were quantified by western blotting. (C) Cells were treated with 1 µM AFM41a in combination with 5 µM 3MA. The phagocytic capacity of cells was measured using pHrodo Red E. coli BioParticles. Data are representative of three independent experiments expressed as means ± SEM. *p < 0.05 vs Control; ***p < 0.001 vs Control; #p<0.05 vs PA; #p<0.001 vs PA; NS, not significant.

Western blot and immunofluorescence analyses consistently demonstrated a significant upregulation of autophagy in THP-1 macrophages treated with AFM32a and AFM41a following PA infection (Figure 4A, B). To further investigate the role of autophagy in AFM41a-affected macrophage polarization, we treated THP-1 macrophages with 3MA and measured the expression levels of iNOS and CD206 using western blotting. The results indicated a notable reduction in iNOS expression upon PA infection in the presence of AFM41a, whereas 3MA treatment significantly reversed the AFM41a-induced reduction in iNOS levels. Conversely, AFM41a-induced increase in CD206 expression was significantly decreased in the 3MA treatment group (**Figure 5B**).

In addition, we quantified the levels of inflammatory cytokines in the cell supernatant using ELISA. The results showed a significant decrease in pro-inflammatory cytokines (TNF-α, IL-6) upon PA infection in the presence of AFM41a, while 3MA treatment markedly increased these cytokine levels that had been reduced by AFM41a. Conversely, the level of the anti-inflammatory cytokine IL-10 was attenuated in the 3MA treatment group compared to the AFM41a treatment group (**Figure 5A**).

Furthermore, we assessed the phagocytic capacity of THP-1 macrophages, which was significantly enhanced upon PA infection in the presence of AFM41a. However, 3MA treatment significantly reduced the phagocytic activity enhanced by AFM41a (**Figure 5C**). These comprehensive findings underscore the pivotal role of autophagy in shaping macrophage polarization and immune responses during PA infection, particularly in the context of PAD2 inhibition.

### Autophagy promotes M2 polarization in PAD2-deficient alveolar macrophages, reducing lung injury in PA-induced sepsis

After establishing the pivotal role of autophagy in alveolar macrophage polarization under PAD2 during PA infection, we expanded our investigation by conducting a comparative analysis between *Pad2^-/^*^-^mice and WT mice. Alveolar macrophages were isolated from both WT and *Pad2^-/^*^-^mice after 24 hours of PA infection. Then the autophagy related protein level was assessed by using western blot and immunofluorescence. The expression level of LC3B, beclin-1, and P62 all suggest *Pad2^-/^*^-^ alveolar macrophages have significantly higher level of autophagy compared to alveolar macrophages of WT mice **(Figure 4C, D)**. Additionally, the number of autophagosomes was measured using TEM, revealing that Pad2-/- alveolar macrophages had higher levels of autophagosomes compared to those in WT mice (Figure S8).

**Figure 6:**
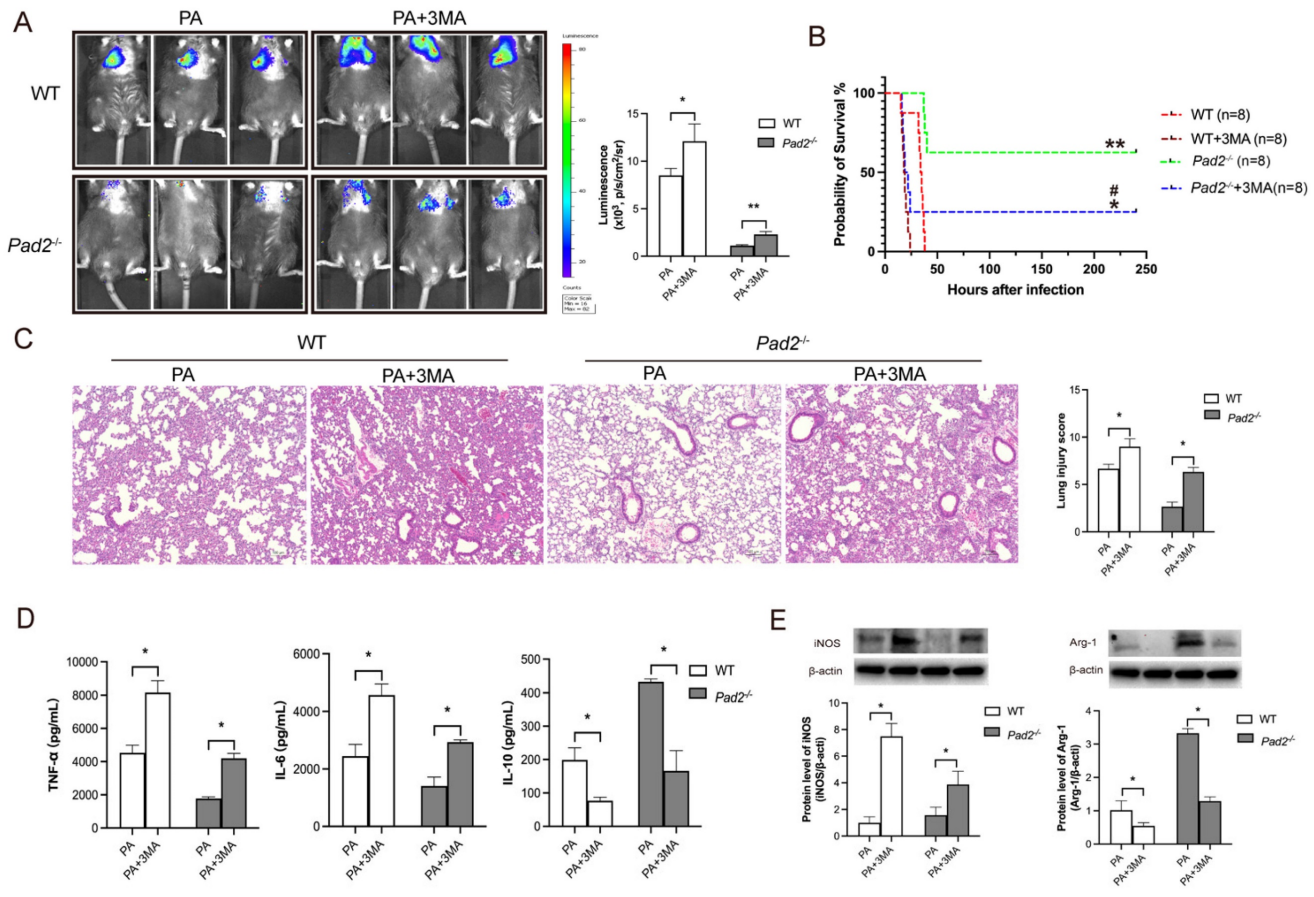
***Pad2* deficiency promoted Alveolar macrophages (AMs) towards M2 polarization through autophagy.** (A) The *Pad2^-/-^* and WT mice were infected with 2.5×10^6^ CFU *Pseudomonas aeruginosa* (PA) Xen-41/mouse, whole animal imaging of bioluminescence was obtained using IVIS XRII system 24 hours after mice challenged with PA. (B) *Pad2^-/-^* and WT mice were infected with 2.5×106 CFU PA 19660/mouse for 24 hours, and 0.5 hours after PA challenge, mice were treated with or without 15mg/kg 3MA. Survival rates were monitored for 10 days (n=8/group), and analysis was conducted using Mantel-Cox test. (C) Lung tissue samples were collected 24 hours after PA infection and subjected to H&E staining. (D) BALF was collected from both groups of mice, with or without 3MA treatment, and the levels of inflammation cytokines (TNF-α, IL-6, and IL-10) were determined using ELISA. (E) AMs from *Pad2^-/-^* and WT mice were lysed, and the expression of iNOS and CD206 were quantified by western blotting. Data are representative of three independent experiments expressed as means ± SEM. *p < 0.05; **p<0.01 vs WT; ***p<0.001 vs WT; ##p < 0.01 vs *Pad2^-/-^*.

To confirm the role of autophagy in sepsis-induced lung injury and the influence on macrophage polarization due to *Pad2* deficiency, we administered the autophagy inhibitor, 3MA, half an hour after PA infection. Notably, 3MA treatment effectively inhibited M2 polarization in alveolar macrophages in both WT and *Pad2^-/^*^-^ mice (**Figure 6E**). Furthermore, it resulted in a broader distribution of bioluminescence in both groups (**Figure 6A**). Importantly, the administration of 3MA significantly decreased the survival rate in PAD2-deficient mice (**Figure 6B**). Histological examination via H&E staining revealed that 3MA treatment increased lung injury that was attenuated by *Pad2* deficiency (**Figure 6C**). These findings were further corroborated by ELISA analysis of pro-inflammatory (TNF-α, IL-6) and anti-inflammatory (IL-10) cytokines in BALF from the lungs of PA-infected WT and *Pad2^-/^*^-^ mice (**Figure 6D**). These results collectively suggest that autophagy plays a crucial role in both lung injury and the modulation of macrophage polarization in the context of *Pad2* deficiency during sepsis induced by PA infection.

## Discussion

Our findings underscore the protective role of *Pad2* deficiency against PA infection, leading to improved survival rates in mice. Notably, we observed a similar enhancement in survival of PA-mice treated with PAD2 inhibitors, with AFM41a demonstrating superior efficacy compared to AFM32a. Our mechanistic investigation unveiled insights into the intricate interplay between PAD2, autophagy, and macrophage polarization, shedding light on potential therapeutic strategies for sepsis management.

Previous studies have highlighted the importance of the PAD2 enzyme in regulating immune responses and cellular processes during infection. Our recent experiments provided compelling evidence that *Pad2* deficiency confers protection against sepsis induced by PA pneumonia 19. In an acute pneumonia model, where PA was intranasally instilled into both *Pad2^-/^*^-^ and WT mice, *Pad2^-/^*^-^ mice exhibited a significantly higher survival rate compared to WT mice. Our *in vivo* imaging studies utilizing PA Xen-41 further revealed that *Pad2* deficiency resulted in a narrower area of bacterial dissemination in the thoracic cavity compared to WT mice, aligning with previous studies implicating PAD2 in regulating the host response to PA infection. To investigate the potential therapeutic implications of targeting PAD2, we evaluated the effects of PAD2 selective inhibitors, AFM32a and AFM41a, on survival rates in PA pneumonia-induced sepsis. Remarkably, both AFM32a and AFM41a treatment improved survival, with AFM41a displaying higher efficacy than AFM32a, highlighting the potential of PAD2 inhibition as a novel therapeutic strategy against PA infection and sepsis.

Macrophages are pivotal players in the innate immune response and contribute to the defense against invading pathogens 27. These immune cells exhibit remarkable phenotypical and functional heterogeneity, capable of modifying their responses based on environmental cues 28, 29, 30. Recent studies have illuminated the significance of macrophage polarization in various diseases, underscoring its pivotal role in disease onset and progression 31-34. We provided evidence that *Pad2* deficiency in PA-challenged mice led to increased presence of macrophages in the BALF compared to PA-challenged WT mice, attributed to a reduction in pyroptosis, a form of inflammatory cell death that contributes to tissue damage and inflammation 19. Our investigation underscores the significant protective effects of PAD2 inhibition using selective inhibitors AFM32a and AFM41a, particularly AFM41a, against PA pneumonia-induced sepsis, as evidenced by the remarkable improvement in survival rates and the notable reduction in bacterial dissemination within the lungs. These results lay the groundwork for future research into the therapeutic potential of targeting PAD2 as a novel strategy to enhance host defense mechanisms and combat severe bacterial infections. Further studies are warranted to unravel the precise molecular mechanisms underlying PAD2-mediated effects in sepsis and validate the translational potential of PAD2 inhibitors as a promising therapeutic approach for infectious and inflammatory diseases.

Autophagy plays a critical role in defending against various infections in metazoan organisms 35-38. Recent studies have reported that autophagy is prominently increased in the early stages of sepsis, exerting its protective role by aiding antigen clearance, inhibiting apoptosis, modulating inflammation, and affecting metabolism. Autophagy emerges as a crucial regulator of macrophage polarization and function 26, 39, influencing immune responses and macrophage polarization through the regulation of inflammatory signaling pathways and cytokine production 39, 40. From our data, *Pad2^-/^*^-^ alveolar macrophages exhibited significantly higher levels of autophagy-related proteins compared to alveolar macrophages from WT mice, suggesting a potential link between *Pad2* deficiency and autophagy regulation upon PA infection. To validate the involvement of autophagy in macrophage polarization caused by *Pad2* deficiency, we administered 3MA, an autophagy inhibitor, to both WT and *Pad2^-/^*^-^ mice half an hour after PA infection. Our results suggest that inhibiting autophagy in *Pad2^-/^*^-^ alveolar macrophages leads to a shift in macrophage polarization towards the M1 phenotype and reduced lung injury in the context of PA infection.

In conclusion, our findings provide valuable insights into the intricate relationship between PAD2, autophagy, and macrophage polarization during PA infection. The heightened autophagy levels in *Pad2*-deficient alveolar macrophages appear to play a critical role in promoting M2 polarization and enhancing bacterial clearance, contributing to the protective effects observed in PA-induced lung injury. Our study highlights the significant impact of *Pad2* deficiency and PAD2 inhibition, particularly the novel PAD2 inhibitor AFM41a, on macrophage polarization during PA infection-induced sepsis. These findings offer valuable insights into the intricate regulation of macrophage responses during bacterial infections and pave the way for potential therapeutic interventions targeting PAD2 and autophagy to modulate macrophage polarization in sepsis management. Further research in this direction could potentially yield novel strategies for enhancing host defense mechanisms and improving patient outcomes in sepsis.

## Supplementary Material

Supplementary figures and table.

## Figures and Tables

**Figure 7:**
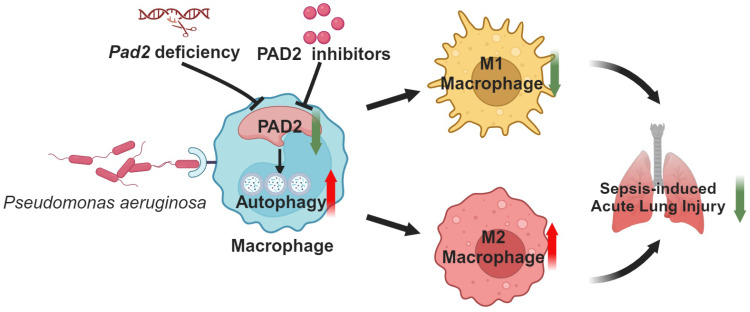
** Schematic mechanism underlying how *Pad2* deficiency or inhibition mitigates *Pseudomonas aeruginosa*-induced lung injury.** Our findings indicate that *Pad2* deficiency or inhibition, particularly with the novel inhibitor AMF41a, not only enhances survival rates and diminishes bacterial proliferation but also enhances macrophage functionality through the augmentation of autophagy in mice.
