# Supplementary material for: AFM41a: A Novel PAD2 Inhibitor for Sepsis Treatment—Efficacy and Mechanism
Source: Int J Biol Sci. 2024 Sep 16;20(13):5043–55. doi: 10.7150/ijbs.97166 (PMC11488583; doi:10.7150/ijbs.97166)
Supplement: Supplementary file 1 — Supplementary figures and table. [file ijbsv20p5043s1.pdf]

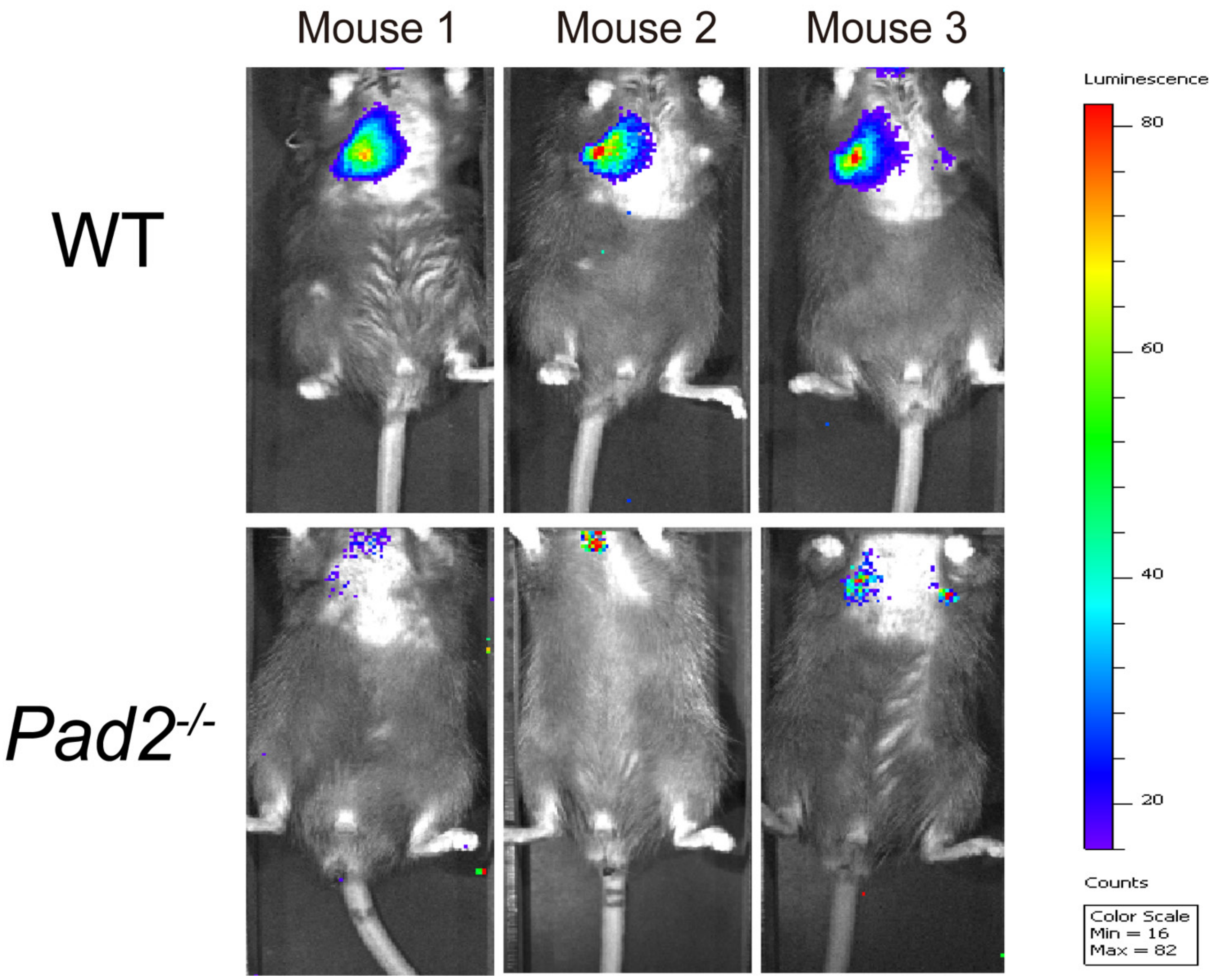

Figure S1. *Pad2* deficiency decreased susceptibility against Pseudomonas aeruginosa (PA) infection.

Pad2$^{-/-}$ and WT mice were intranasally challenged with 2.5×10^6 CFU PA 19660/mouse. Whole animal imaging of bioluminescence was obtained using IVIS XRII system 24 hours after mice challenged with 2.5×10^6 CFU PA Xen-41/mouse.

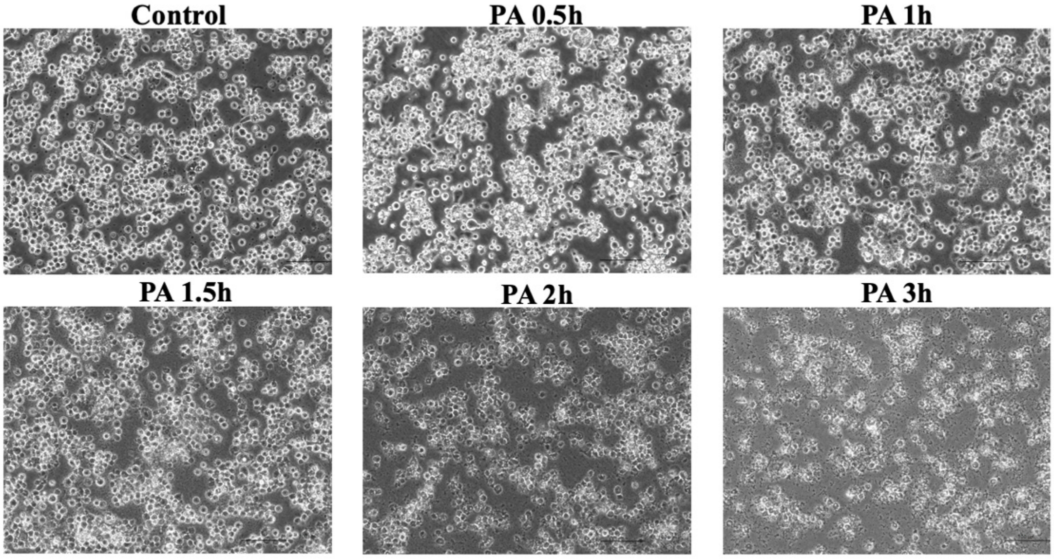

Figure S2. Effect of Pseudomonas aeruginosa (PA) infection on Morphology of THP-1 derived macrophages.

THP-1 cells were treated with PMA to induce differentiation for 22–24 hours prior to infection with PA 19660 at a multiplicity of 100. At the time points indicated, phase contrast images of cultures were recorded.

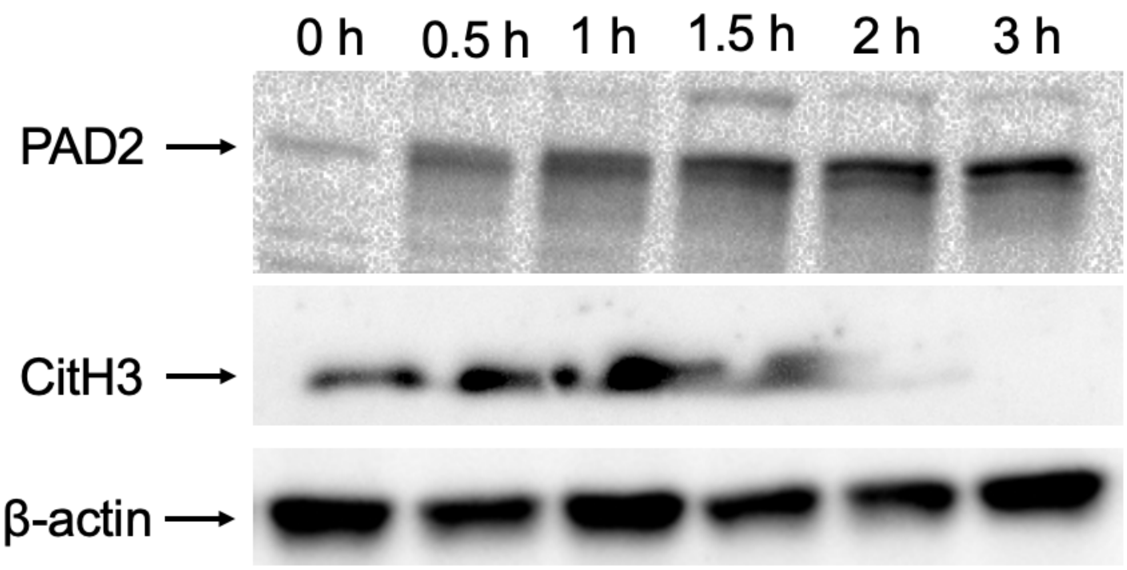

Figure S3. Effect of Pseudomonas aeruginosa (PA) infection on protein expression of PAD2 and CitH3 in THP-1 derived macrophages.

THP-1 cells were treated with PMA to induce differentiation for 22–24 hours prior to infection with PA 19660 at a multiplicity of 100. Protein expression levels of PAD2 and CitH3 were measured at different time points using western blot analysis.

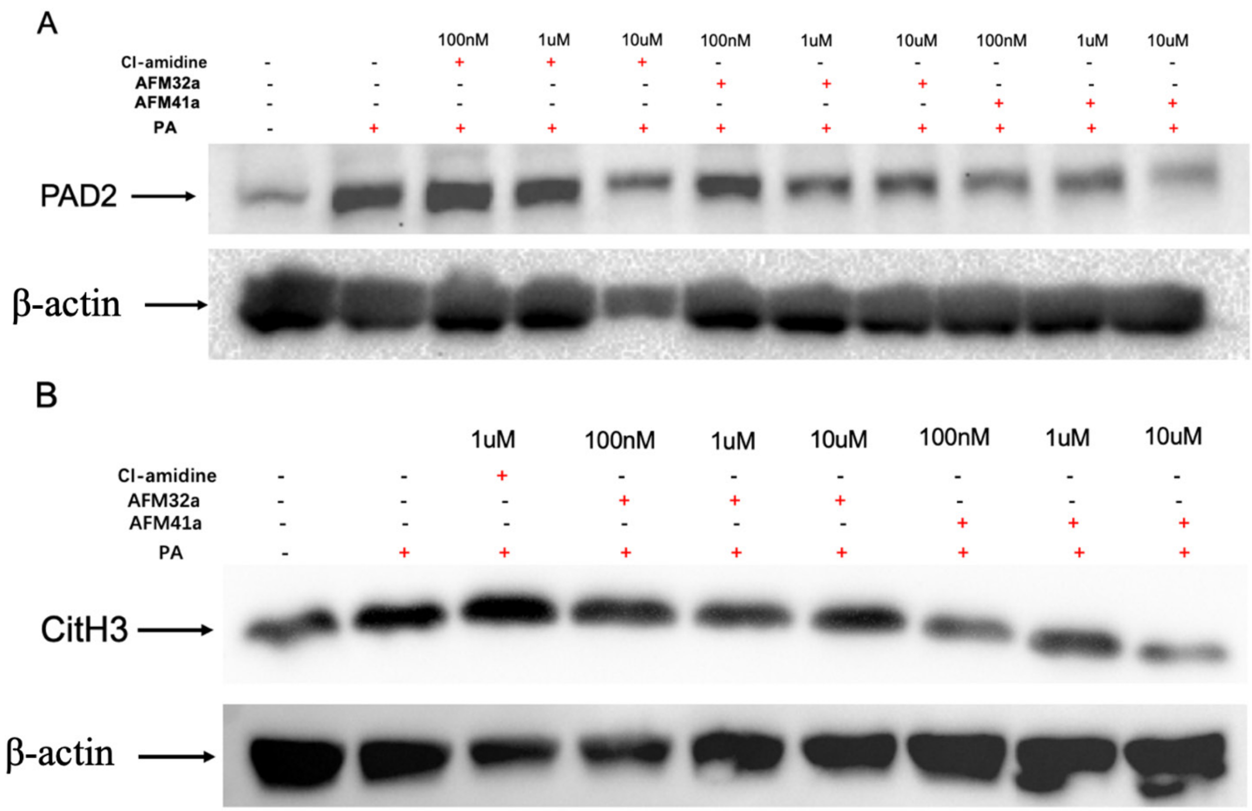

Figure S4. Effect of PAD inhibitors on protein expression of PAD2 and CitH3 in THP-1 derived macrophages.

THP-1 cells were treated with PMA to induce differentiation for 22–24 hours prior to infection with PA 19660 at a multiplicity of 100. Cells were then pre-treated with Cl-amidine, AFM32a and AFM41a at different concentrations for 24 hours, then treated with PA for 1 hour. Protein expression levels of PAD2 (A) and CitH3 (B) were measured using Western blot analysis.

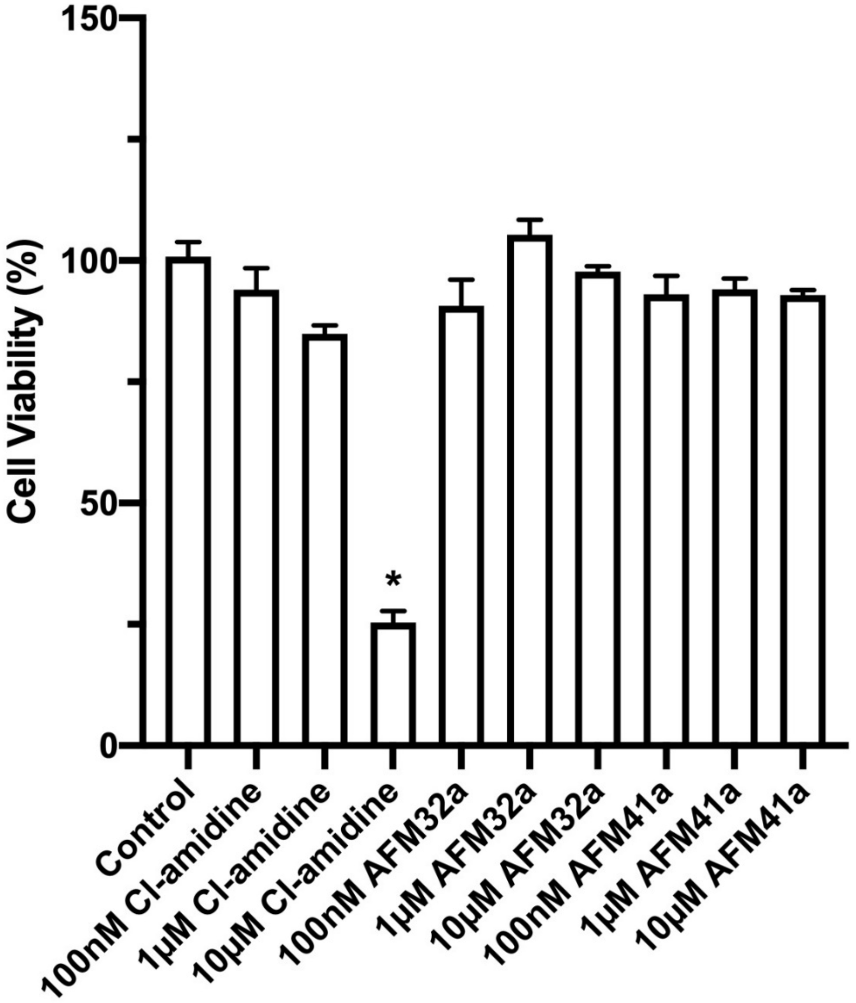

Figure S5. Effect of PAD inhibitors on cell viability in THP-1 derived macrophages.

THP-1 cells were treated with PMA to induce differentiation for 22–24 hours prior to be treated with three different concentrations of Cl-Amidine, AFM32a, and AFM41a for 24 hours, then cell viability was assessed by MTT assay. Data are representative of three independent experiments expressed as means ± SEM. $^{*}p < 0.05$.

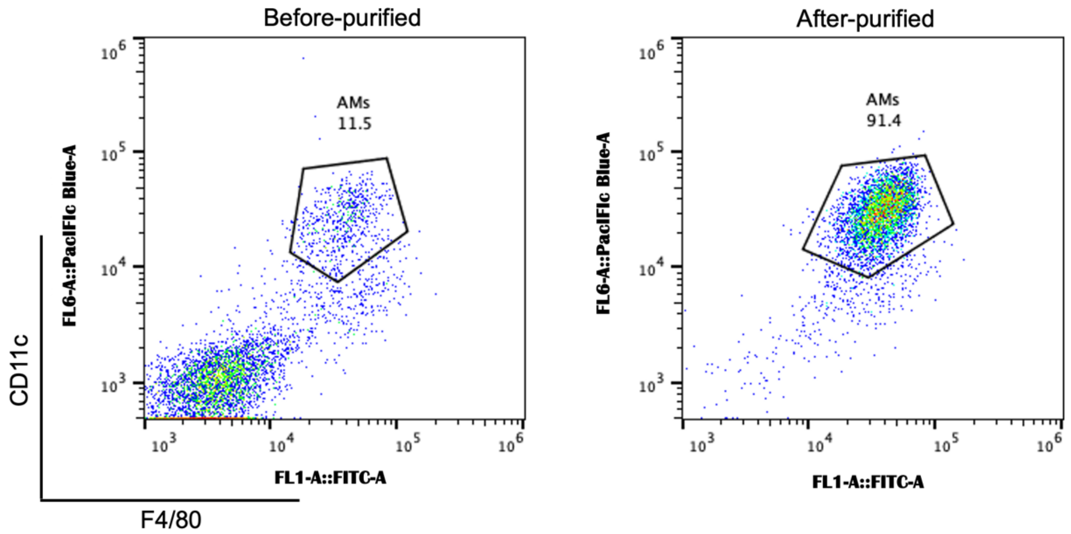

Figure S6. Analyses of purified alveolar macrophages (AMs) from bronchoalveolar lavage fluid.

The WT mice were infected with 2.5×10^6 CFU PA 19660/mouse for 24 hours. Alveolar cells were then isolated and cultured in RPMI 1640 medium for 1.5 hours to purify and collect AMs. Both the isolated and purified cells were subsequently subjected to flow cytometry analysis to quantify the distribution of macrophage populations.

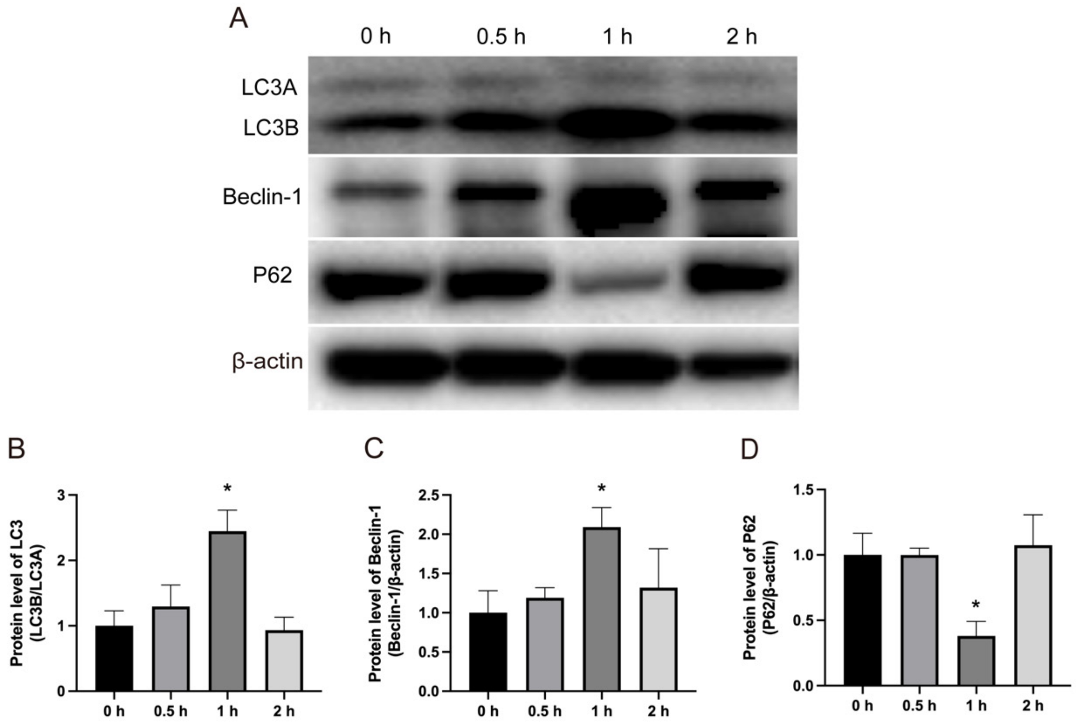

Figure S7. Effect of Pseudomonas aeruginosa (PA) infection on expression of autophagy proteins in THP-1 derived macrophages.

THP-1 cells were treated with PMA to induce differentiation for 22–24 hours prior to infection with PA 19660 at a multiplicity of 100. Protein expression levels of LC3, Beclin-1 and P62 were measured at different time points using western blot analysis. Data are representative of three independent experiments expressed as means ± SEM. *$p < 0.05$ vs 0h.

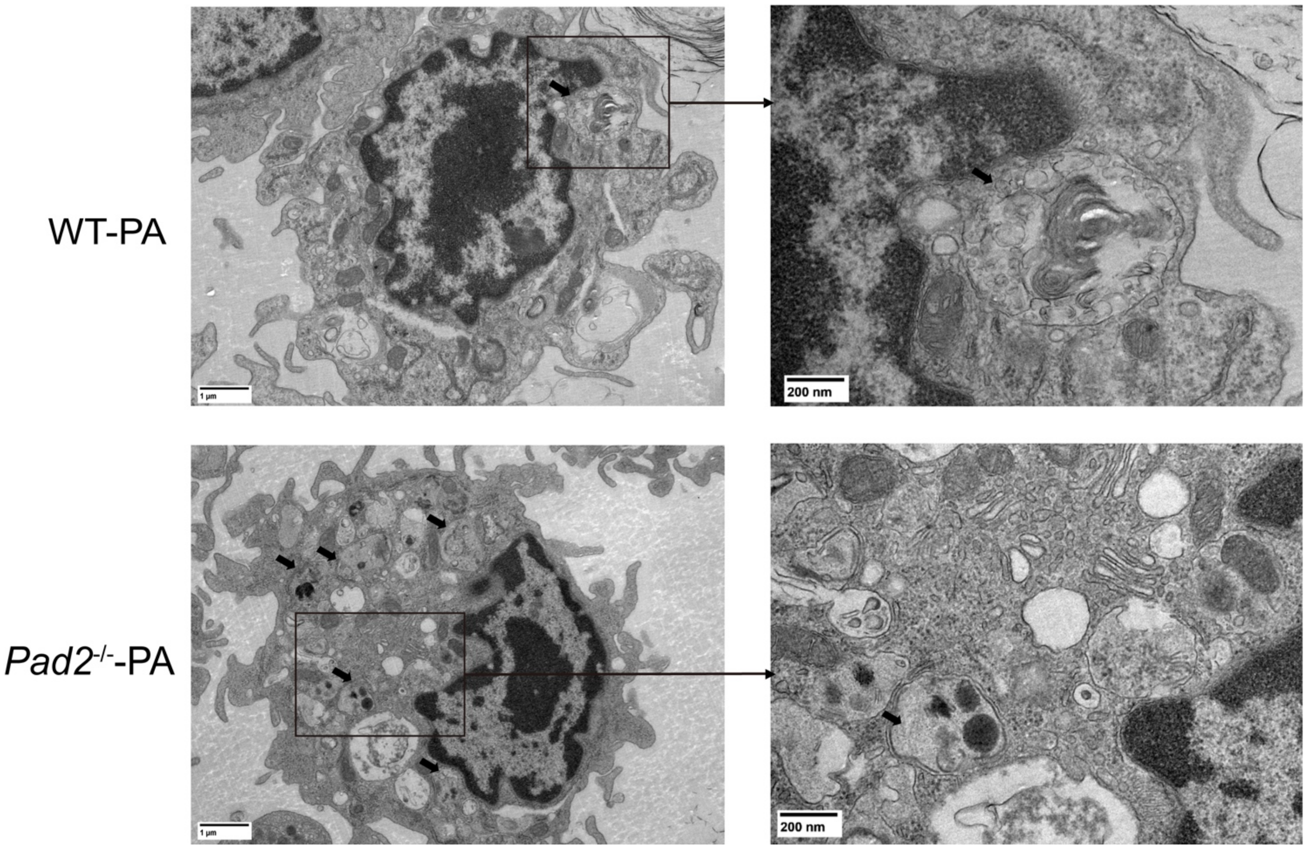

Figure S8. ***Pad2*** **Deficiency Increases Autophagosome Formation.**

Pad2$^{-/-}$ and WT mice were infected with 2.5×10^6 CFU of PA 19660 per mouse for 24 hours. Macrophages were collected from the bronchoalveolar lavage fluid of the mice 24 hours post-PA infection. The cells were subsequently analyzed using transmission electron microscopy (TEM) to observe the presence of autophagosomes, which are indicated by solid arrows.

Table S1 List of genes and primers used for qRT-PCR.

Gene Symbol	Primers (5’→3’) (F=forward, R=reverse)
Tnfα (mouse)	F: 5′-TCTTCTCATTCCTGCTTGTGG-3′<br>R: 5′-GGTCTGGGCCATAGAACTGA-3′
Il6 (mouse)	F: 5′-GTTCTCTGGGAAATCGTGGA-3′<br>R: 5′-GGAAATTGGGGTAGGAAGGA-3′
Il10 (mouse)	F: 5′-CCAAGCCTTATCGGAAATGA-3′<br>R: 5′-TTCTCACCCAGGGAATTCAA-3′
Nos2 (mouse)	F: 5′-CAGCTGGGCTGTACAAACCTT-3′<br>R:5′-CATTGGAAGTGAAGCGTTTCG -3′
Ccl2 (mouse)	F: 5′-GCAGCAGGTGTCCCAAAGAA-3′<br>R: 5′-GGTCAGCACAGACCTCTCTCTTG-3′
Arg1 (mouse)	F: 5′-CTCCAAGCCAAAGTCCTTAGAG-3′<br>R: 5′-AGGAGCTGTCATTAGGGACATC-3′
Mrc1 (mouse)	F: 5′-CTAACTGGGGTGCTGACGAG-3′<br>R:5′-GGCAGTTGAGGAGGTTCAGT-3′
Gapdh (mouse)	F: 5′-TGGCATTGTGGAAGGGCTCATGAC-3′<br>R: 5′-ATGCCAGTGAGCTTCCCGTTCAGC-3′
Tnfα (human)	F: 5′- CCTCTCTCTAATCAGCCCTCTG-3′<br>R: 5′-GAGGACCTGGGAGTAGATGAG-3′
Il6 (human)	F: 5′-ACTCACCTCTTCAGAACGAATTG-3′<br>R: 5′-CCATCTTTGGAAGGTTCAGGTTG-3′
Il10 (human)	F: 5′-TACGGCGCTGTCATCGATT-3′<br>R: 5′-GGCTTTGTAGATGCCTTTCTCTTG -3′
Nos2 (human)	F: 5′-CGGTGCTGTATTTCCTTACGAGGCGAAGAAGG-3′<br>R: 5′-GGTGCTGCTTGTTAGGAGGTCAAGTAAAGGGC-3′
Ccl2 (human)	F: 5′-GATCTCAGTGCAGAGGCTCG-3′<br>R: 5′-TTTGCTTGTCCAGGTGGTCC-3′
Mrc1 (human)	F: 5′-CTACAAGGGATCGGGTTTATGGA-3′<br>R: 5′-TTGGCATTGCCTAGTAGCGTA-3′
Gapdh (human)	F: 5′-CCACTCCTCCACCTTTGAC-3′<br>R: 5′-ACCCTGTTGCTGTAGCCA-3′
